# Induction of Plasmodium-Specific Immune Responses Using Liposome-Based Vaccines

**DOI:** 10.3389/fimmu.2019.00135

**Published:** 2019-02-01

**Authors:** Aloysious Ssemaganda, Ashwini Kumar Giddam, Mehfuz Zaman, Mariusz Skwarczynski, Istvan Toth, Danielle I. Stanisic, Michael F. Good

**Affiliations:** ^1^Institute for Glycomics, Griffith University, Southport, QLD, Australia; ^2^School of Chemistry and Molecular Biosciences, The University of Queensland, Brisbane, QLD, Australia; ^3^School of Pharmacy, The University of Queensland, Brisbane, QLD, Australia; ^4^Institute for Molecular Bioscience, The University of Queensland, Brisbane, QLD, Australia

**Keywords:** malaria, *Plasmodium*, immunity, adjuvant, liposomes, vaccine

## Abstract

In the development of vaccines, the ability to initiate both innate and subsequent adaptive immune responses need to be considered. Live attenuated vaccines achieve this naturally, while inactivated and sub-unit vaccines generally require additional help provided through delivery systems and/or adjuvants. Liposomes present an attractive adjuvant/delivery system for antigens. Here, we review the key aspects of immunity against *Plasmodium* parasites, liposome design considerations and their current application in the development of a malaria vaccine.

## Introduction

Malaria vaccine development has been a focus of research since the 1940s when inoculation with homologous inactivated sporozoites and/or serum resulted in control of parasitemia amongst immunized domestic fowls (1, 2). Follow-up studies also showed that monkeys and ducks were protected against *Plasmodium (P) knowlesi* and *P. lophurae* following vaccination with killed, adjuvanted parasites (3, 4). Additionally, in 1967, it was demonstrated for the first time, that immunization with irradiated sporozoites protected mice against *P. berghei* (5). Despite these early promising findings, an effective vaccine for malaria still eludes scientists with only one vaccine candidate, RTS, S, receiving a positive scientific opinion from European regulators and it is currently approved for use in pilot implementation trials in 3–5 epidemiologically distinct locations in sub-Saharan Africa (6, 7). The complexity of *Plasmodium* parasites, antigenic polymorphism and failure to maintain long-lived immune responses calls for continued efforts in the search for novel vaccines which can effectively prevent *P. falciparum* and *P. vivax* infections (8).

A major bottleneck in the development of vaccines against infectious diseases is the failure to initiate robust innate immune responses and subsequent potentiation and maintenance of downstream adaptive immune responses. This is achieved naturally with live attenuated vaccines while inactivated and sub-unit vaccines require delivery systems and/or adjuvants for efficient presentation to the immune system and additional stimulation to enhance potency (9). To address this, careful selection of adjuvants and delivery systems needs to be considered early in the vaccine development process. However, only a handful of adjuvants have been licensed or tested for use in human vaccines and these are summarized in Table 1.

**Table 1:** Clinically tested and licensed vaccine adjuvants (10, 11).

**Adjuvant**	**Status of vaccine adjuvant**
Aluminum-based salts (Alum)	Licensed for tetanus, diphtheria, pneumococcus vaccines (12).
Mf59 (an oil-in-water emulsion consisting of squalene, Tween 80 and span 85)	Licensed for influenza vaccines (13, 14).
AS01: Liposome-based adjuvant comprised of Monophosphoryl Lipid A (MPLA) and QS-21	Tested in phase III malaria and shingles vaccine trials (15, 16).
AS02: squalene emulsion comprised of MPLA and QS-21	Tested in phase II malaria trials (17).
AS03: An oil-in-water emulsion comprised of squalene, Tween 90 and α-tocopherol	Licensed for influenza vaccines (18, 19).
AS04: comprised of aluminum hydroxide and MPLA	Licensed for Cervarix vaccine against HPV and Fendrix against hepatitis B (20–22).
ISA-51 Montanide: Mineral oil with a Mannide monooleate emulsifier	Licensed for Cimavax vaccine against non-small cell lung cancer (NSCLC) (23).
Virosomes: Comprised of influenza virus envelopes reconstituted in liposomes	Licensed for hepatitis A (Epaxal) (22, 24) and Influenza vaccines (Invivac, Inflexal) (25).
CAF01: Cationic liposomes comprised of dimethyldioctadecylammonium bromide (DDAB) and trehalose 6,6-dibehenate (TDB)	Tested in phase I HIV and tuberculosis vaccine trials (26, 27).
IC31: TLR9 agonist	Tested in phase I tuberculosis vaccine trials (28–30).
Poly I:C: TLR3 agonist comprised of repeating units of double stranded inosine and cytosine	Tested in phase I/II cancer vaccine trials (31).
Imiquimod: TLR7/8 agonist	Tested in phase II therapeutic vaccine trials against vulval intraepithelial neoplasia (32)
SE/SE-GLA: Squalene emulsion co-formulated with TLR4 agonist GLA	Tested in phase I influenza vaccine trials (33–36)
ISCOMS, ISCOMATRIX/ Matrix-M™: Lipid-based adjuvants formulated with cholesterol and saponins	Tested in a phase I vaccine trials against HCV, HPV and influenza (33–35, 37) (NCT02905019)
Recombinant CTB: B subunit of cholera toxin	Licensed for the cholera vaccine, Dukoral (38)

First proposed by Gregoriadis and Allison in 1974 as immunological adjuvants (39), liposomes are a promising vaccine adjuvant/antigen delivery system. Historically well-known as drug carriers, liposomes are self-assembling phospholipid vesicles capable of incorporating and protecting antigens from degradation, as well as facilitating antigen delivery to professional antigen presenting cells (APCs) (9, 40–43). Liposomes generally act by depot formation resulting in enhanced uptake by APCs and subsequent induction of the desired immune responses. To date, the extensive use of liposomes can be attributed to their safety profile, biocompatibility, biodegradability, versatility, and plasticity and therefore they present an attractive platform for malaria vaccine development.

In a natural malaria infection, the acquisition of clinical immunity is slow, spanning several years of repeated exposure, and it is not sterile (44). An ideal vaccine capable of inducing sterile immunity against the different life-cycle stages of malaria will need to induce a qualitatively and/or quantitatively different immune response to that induced during natural infection immunity (45). Since stage-specific immunity to malaria requires humoral and cell-mediated immune responses, the ideal vaccine-induced responses should preferably be comprised of both forms of responses. However, for rational vaccine development, a clear understanding of the complex nature of immunity to malaria is required and this in turn may help to inform the selection of an appropriate adjuvant/delivery system. This review highlights key aspects of the immune response to malaria, design considerations of liposomes, and their current application in malaria vaccine development.

## Immunity to Malaria

*Plasmodium* parasites, the causative agents of malaria, are obligate intracellular organisms which undergo a complex life-cycle in the vertebrate host broadly divided into: the mosquito stage which occurs in the vector; the pre-erythrocytic stage which occurs in the vertebrate host's liver; and the erythrocytic stage which occurs in the blood of the vertebrate host (46). At all life-cycle stages, the immune responses induced following infection differ significantly and a clear understanding of these responses will inform the vaccine development process.

### Pre-erythrocytic Stage Immunity to Malaria

The pre-erythrocytic stage of malaria infection is clinically quiescent, probably due to the low number of sporozoites inoculated by the mosquito while taking a blood meal. During this stage, studies in mice have shown that antibodies can control infection through immobilization of sporozoites by inhibiting sporozoite motility and subsequent invasion of hepatocytes (47, 48). Following natural infection, studies demonstrated the existence of pre-erythrocytic antigen-specific antibodies to *P. falciparum*; however, their role remains unclear (49–51). To date, the best model that has enabled the study of pre-erythrocytic immune response mechanisms has utilized irradiated sporozoites in both humans and animals. Radiation-attenuated sporozoites retain the capacity to infect hepatocytes but cannot develop into an erythrocytic infection. Studies in rodent models involving inoculation of radiation-attenuated sporozoites demonstrated that antibodies were involved in the enhanced clearance of sporozoites, reduction in sporozoite motility and inhibition of hepatocyte invasion (52, 53). In clinical studies, induction of antibodies to the circumsporozoite protein (CSP) following immunization with the *P. falciparum* sporozoite (PfSPZ) vaccine has been shown to partially correlate with protection (54–58). Furthermore, following immunization with RTS, S/AS01, high CSP-specific antibody titers are induced and are a surrogate measure of protective efficacy for this vaccine candidate (16, 59–63).

Cell-mediated immunity following inoculation of radiation-attenuated sporozoites has also been shown to contribute to vaccine-induced sterilizing immunity to *Plasmodium* infection in both mice and humans (54–58, 64–72). Studies in rodent models, however, indicate that a network of cellular mechanisms mediates immunity to pre-erythrocytic infection (64–66, 68, 70–72). Initial studies in mice immunized with irradiated *P. yoelii* 17XNL sporozoites reported that cytotoxic CD8^+^ T cells mediated protection against a wild-type challenge of infectious sporozoites (64, 65). Further studies indicated that IL-12 from liver antigen presenting cells (APCs) stimulated CD8^+^ T cells and Natural killer (NK) cells to produce interferon (IFN)-γ. IFN-γ, in turn, induces the infected hepatocytes to produce nitric oxide (NO), which subsequently kills the parasite in the hepatocyte (66, 68, 71). In addition, the balance between IL-2, IL-10, IL-12, and IFN-γ results in an inflammatory response that contributes to pre-erythrocytic immunity (70, 72). In human volunteers, both CD4^+^ and CD8^+^ T cell responses against pre-erythrocytic antigens have been observed following immunization with irradiated sporozoites (54–58, 67, 69).

Controlled infection immunization (CII) studies, involving experimental sporozoite inoculation via infectious mosquito bites with concurrent chemoprophylaxis provides another model for investigating pre-erythrocytic immunity to malaria. These studies have shown that a protective polyfunctional T cell response to pre-erythrocytic antigens predominantly characterized by the production of IFN-γ, tumor necrosis factor (TNF), and IL-2 is induced following CII (73–76). Purified IgG against *P. falciparum* sporozoites, obtained from CII trial participants was also shown to inhibit liver stage infection in a humanized liver-chimeric mouse model (73, 75, 77).

### Erythrocytic Stage Immunity to Malaria

During the erythrocytic stage, clinical signs, and symptoms of malaria manifest as the parasites invade and replicate in RBCs. Studies in animal models have demonstrated that the innate immune system is involved in the initial recognition of blood-stage parasites, promotion of inflammation, inhibition of parasite growth and potentiation of the adaptive immune response (78, 79). *Plasmodium falciparum* pathogen-associated molecular patterns (PAMPs) such as glycosylphosphatidylinositol (GPI) anchors (Toll-like receptor 2 [TLR2]), hemozoin (NOD-like receptor containing pyrin domain 3 [NLRP3] inflammasome), CpG-containing DNA motifs bound to hemozoin (TLR9) and AT-rich DNA motifs (unidentified cytosolic receptor) have been shown to trigger an inflammatory cascade by binding pattern recognition receptors (PRRs) on the surface of innate immune cells (79–83). This interaction of PAMPs and PRRs results in the production of pro-inflammatory cytokines (IL-12 [p70], IFN-γ and TNF) by APCs, as well as regulatory cytokines (IL-10 and TGF-β) that have been implicated in immunity and pathogenesis to blood-stage malaria infection (79, 84, 85).

Several innate immune cells such as dendritic cells (DCs), macrophages, mast cells, neutrophils, NK cells, natural killer T (NKT) cells, and γδ T cells have been implicated in this initial immune response (85–88). *Plasmodium* parasites have been shown to modulate DC maturation and function resulting in the induction of regulatory T cells which in turn modulates CD4^+^ T cell responses, suppressing protective immune responses while averting immune-mediated pathology (85). IL-12 production by DCs has also been implicated in the production of IFN-γ by NK cells and CD4^+^ Th1 cells resulting in the control of parasite growth (85). Understanding the balance between protective and immunopathologic responses following DC activation and maturation might have favorable implications in designing vaccines to prevent severe malaria (85). *In vitro*, NK cells have been shown to be an early source of IFN-γ, promoting the destruction of infected red blood cells by activated macrophages (79, 89). γδ T cells and monocytes on the other hand have been associated with elevated levels of TNF, IL- 10, IP-10, IL-6, macrophage inflammatory protein (MIP)-1β and MIP-1α which is linked with severe disease (90). Furthermore, downregulation of γδ T cell responses following repeated exposures to *Plasmodia* has also been implicated with better tolerance to clinical malaria (91, 92). Given their specificity for restricted TCR ligands, γδ T cells present an attractive target for a vaccine to protect against severe disease (45, 90).

Antibodies play a role in naturally acquired immunity to erythrocytic stage malaria, as it has been shown that the passive transfer of immunoglobulin from immune donors resulted in the reduction of parasitaemia and clinical disease among semi-immune recipients from East Africa as well as non-immune Thai patients (93–95). Antibodies may function by inhibiting merozoite invasion of RBCs (96), binding to pRBCs and enhancing clearance by the spleen (97, 98), as well as opsonizing pRBCs, resulting in phagocytosis by macrophages (99, 100).

Cell-mediated immunity against the erythrocytic stage is primarily mediated by CD4^+^ T cells, as demonstrated in both murine and human models. Studies showed that mice depleted of CD4^+^ T cells developed very high parasitaemia and were unable to control the infection compared to mice depleted of CD8^+^ T cells, which developed mild parasitaemia that subsequently resolved; this indicated a clear role of CD4^+^ T cells in erythrocytic stage immunity (101, 102). Additionally, adoptive transfer of CD4^+^ T cells was shown to confer protection and control parasitaemia in immunodeficient mice (103). Further investigation of the role of CD4^+^ T cells demonstrated that during the acute phase of infection, there was a significant upregulation of an IFN-γ-specific CD4^+^ T cell (Th1) response followed by an IL-4-specific CD4^+^ T cell-mediated (Th2) antibody response during the chronic phase (104). These data indicate that early activation of Th1 cells enables control of the infection via effector mechanisms such as macrophages, followed by a Th2 response which activates B cells to clear the parasite in the later stages of the infection in mice (105, 106).

In human studies, the role of CD4^+^ T cells was demonstrated when volunteers were infected with low doses of blood-stage *P. falciparum* followed by drug cure (107). In this study, volunteers appeared to be protected against a homologous challenge infection with immunity associated with an IFN-γ-specific CD4^+^ T cell response and nitric oxide synthase (NOS) production in the absence of detectable antibodies (107). However, a follow-up study suggested that residual drug may have contributed to the apparent protection (108). Another study showed that stimulation of T cells obtained from children living in Papua New Guinea resulted in parasite-specific IFN-γ and TNF responses, which were associated with protection against clinical episodes of malaria (109). More recently, studies in African children showed that CD4^+^ T cells may play an important modulatory role in the development of blood-stage immunity (110, 111). Higher IFN-γ/IL-10+ specific-CD4^+^ T cell responses were observed amongst children heavily exposed to malaria compared to children with low exposure indicating that CD4^+^ T cells may play an important immunomodulatory role in the pathogenesis of childhood malaria (110). Additionally, the induction of IL-10-producing CD4^+^ T cells amongst highly exposed children may interfere with the development of immunity, which may have implications for vaccine development (111).

T follicular helper cells (Tfh) are a subset of CD4^+^ T cells, capable of providing B cell help as well as activating follicular B-cell responses (112–114). Recent studies in mice showed that Tfh cells play a critical role in controlling *P. chabaudi* blood-stage infection via activation of IL-21 mediated responses (115). Therefore, since humoral responses are critical to the erythrocytic stages of *Plasmodium*, an in-depth understanding of the activation and maintenance of Tfh cells during malaria will be critical in designing blood-stage vaccines (115).

Regulatory T cells (Tregs) are another CD4^+^ T cell subset implicated in the maintenance of immune homeostasis and control of excessive pathogen-driven inflammatory responses (116, 117). Following *Plasmodium* infection, uncontrolled production of pro-inflammatory cytokines is associated with pathology in both mice and humans (118–121) and anti-inflammatory cytokines (TGF-β and IL-10) are known to be critical in the modulation of this inflammatory response (118, 120, 122, 123). The immunomodulatory function of IL-10 and TGF-β is associated with Tregs whose role in rodent malaria remains unclear. Some studies have shown that Tregs are critical in the control of pro-inflammatory responses associated with pathology (124, 125) while other studies have associated upregulation of Tregs with detrimental outcomes (124, 126, 127). These discrepancies may have been dependent on the rodent parasite strains utilized in the study (128). Tregs may inhibit protective immune responses resulting in enhanced parasite growth if induced early in infection but may also limit immune-mediated pathology (45, 128–130). Clinical studies have shown that acute infection with *Plasmodium* parasites resulted in upregulation of Tregs which positively correlated with augmented parasite load and subsequent disease severity amongst these individuals (131–134). Given this background, a vaccine capable of inducing Tregs with the ability to protect against the immunopathology associated with malaria infection would be desirable if parasite persistence is required for the maintenance of protective immune responses. However, since Tregs are known to inhibit protective immune responses, treatment with antimalaria drugs at the time of vaccination may be necessary to “normalize” the pre-existing immune response and ensure induction of the appropriate vaccine-specific responses (45).

The role of CD8^+^ T cells in defense against blood-stage parasites remains unclear due to the fact that mature RBCs do not express MHC class I molecules. However, *in vitro* studies have shown that both *P. falciparum* and *P. vivax* are able to invade erythroblasts—immature erythrocytes that possess a nucleus and express MHC class I molecules (135, 136). Additionally, studies in mice demonstrated that blood-stage parasite antigens were cross-presented by CD8-α^+^ DCs, inducing parasite-specific CD8^+^ T cell responses capable of lysing APCs (137). These findings indicated the possible relevance of CD8^+^ T cells in erythrocytic immunity to *Plasmodium*. Indeed, additional studies in mice demonstrated that parasitized erythroblasts activated CD8^+^ T cells in an antigen-specific manner (138). This contact-dependent Fas—Fas Ligand (FasL) interaction of CD8^+^ T cells with the parasitized erythroblasts results in the exposure of phosphatidylserine (PS) on the erythroblast surface (139). Cells displaying PS on their surface are rapidly phagocytosed by macrophages. Thus, CD8^+^ T cells in conjunction with macrophages are able to mediate immunity to a blood-stage malaria infection in mice (139). Earlier studies in a *P. yoelii* infection model demonstrated that professional APCs might cross-present parasite-derived peptides on MHC class I to CD8^+^ T cells leading to cytotoxicity through the production of IFN-γ, perforin and granzyme B (140). Furthermore, parasite-specific CD8^+^ T cells have also been shown to clear infected reticulocytes, which express MHC class I molecules, via the secretion of IFN-γ and expression of granzyme B (141).

These data highlighted in the studies above provide an insight into the complex nature of immune responses elicited following infection with malaria parasites. Understanding the balance between protective immunity and immunopathology is critical for the development of an ideal vaccine capable of inducing both humoral and cell-mediated immune responses against different life-cycle stages of the malaria parasite. To achieve this balance, careful selection of antigen delivery systems and adjuvants during the vaccine development process is paramount and the pliability of liposome-based platforms can be utilized for this purpose.

## Design Considerations of Liposomal Vaccine Formulations

**Figure 1:**
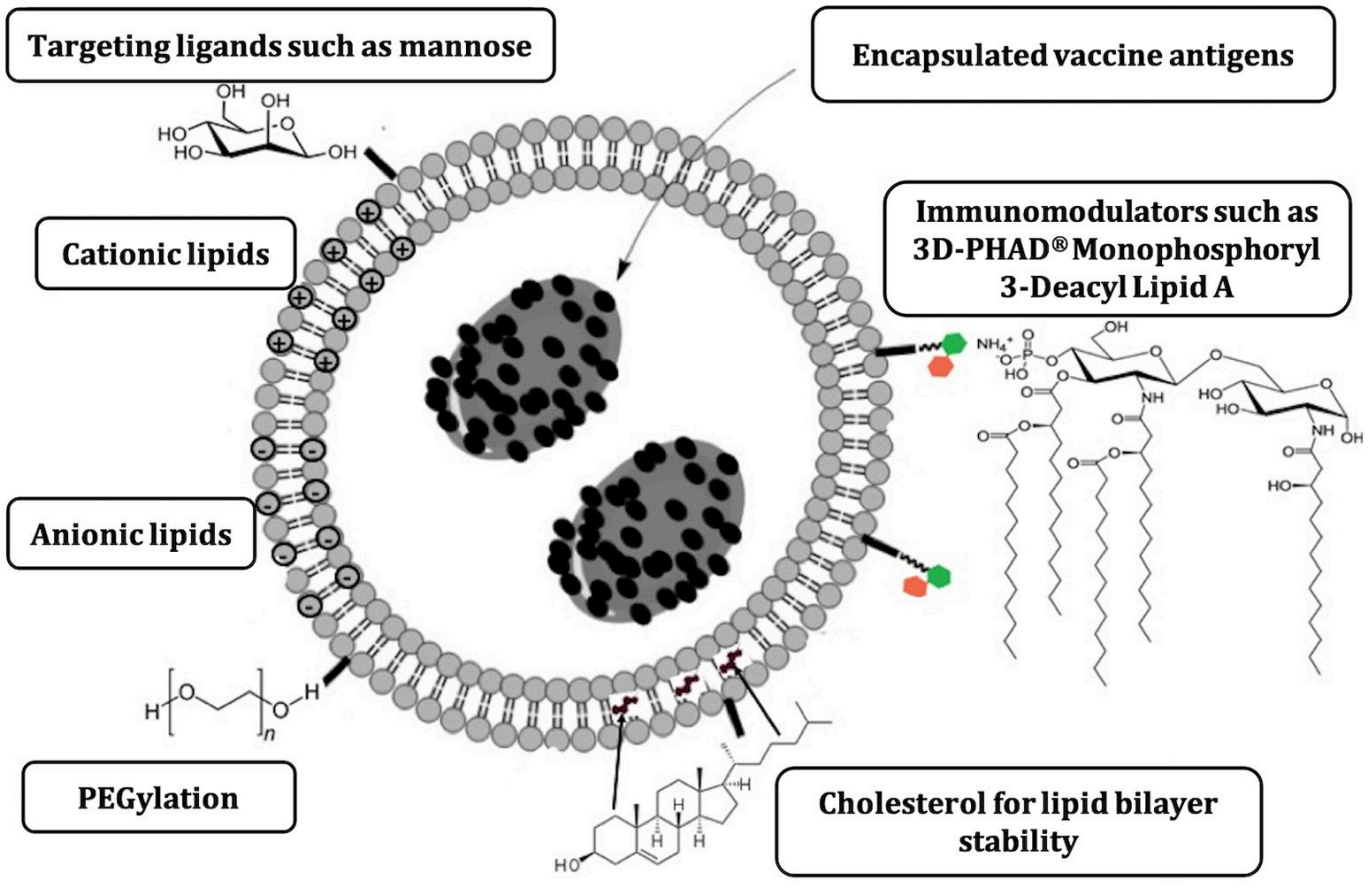
Major liposome physicochemical characteristics that can be modified to direct immune responses elicited following vaccination (9).

Based on their design, liposome vaccine formulations can be tailored to achieve desired immune responses and adjuvant properties by modification of vesicle physicochemical factors (summarized in Figure 1), such as lipid composition, charge, PEGylation, antigen encapsulation, and addition of immunomodulators (9, 40–43).

The choice of phospholipid has been shown to enhance the adjuvanticity of liposomes. Long chain lipids tend to form rigid ordered bilayer structures while those with shorter tails tend to form fluid and disordered vesicles (9). Immunization of animals with liposomes formulated with long chain lipids such as dimethyldioctadecylammonium bromide (DDAB) and distearyol derivative of L-α-phosphatidyl choline (DSPC) resulted in stronger antigen-specific antibody responses when compared to animals that received liposomes formulated with shorter chain lipids (142). Additionally, the stability of the lipid bilayer can be enhanced further with the addition of cholesterol within the liposome formulation resulting in improved antibody responses compared to formulations without cholesterol (143–145).

Positively charged (cationic) liposomes formulated with saturated acyl chain lipids (with a quaternary ammonium head group) have been shown to promote the binding of antigen at the site of injection stimulating interaction with APCs to elicit a robust Th1 cytokine response (146). In contrast, highly fluid unsaturated acyl chain liposomes that are rapidly cleared from the injection site result in lower activation of APCs as measured by the decreased expression of DC co-stimulatory molecules CD40 and CD86 (146). Additionally, cationic liposomes have been shown to promote retention of higher levels of antigen at the injection site, resulting in a depot-effect allowing continuous attraction of APCs and subsequent induction of robust cell-mediated immune responses comprised of IFN-γ, IL-2, TNF, and IL-17 (147). On the other hand, negatively charged (anionic) and neutral liposomes were rapidly cleared from the injection site resulting in lower activation of APCs and lower Th1 as well as Th17 cytokine responses (9, 147, 148).

The inclusion of polyethylene glycol (PEG), known as PEGylation, has been extensively used for the stabilization of liposomes (149). PEGylation has been shown to mask the charge, due to the hydrophilic chains of PEG extending out from the surface of the liposomes subsequently reducing the electrostatic retention of antigen to the surface of these vesicles (150, 151). Additionally, PEGylation influences lipid packaging reducing the number of bilayers and resulting in reduced vesicle size (151). This modification of liposome size and antigen adsorption properties by PEG was shown to reduce depot formation, resulting in reduced IgG2b antibody and Th1 (IFN-γ) cytokine responses as well as an elevated Th2 (IL-5) cytokine response compared to non- PEGylated liposome formulations (150, 151).

The particle size of liposomes has also been shown to impact adjuvanticity and direct the development of the resulting cell-mediated immune response (42). Studies have shown that the immune response induced following administration of small-sized liposome vesicles (10–100 nm) was skewed toward Th2 whilst larger vesicles (400–2,500 nm) induced a Th1 response characterized by augmented IFN-γ and IgG2a production (152). The differences in the profiles of the induced immune response of large vs. small vesicles could be due to differences in antigen processing and trafficking to lymph nodes. Large-sized vesicles (560 nm) were shown to be more efficiently phagocytosed and processed by macrophages compared to smaller vesicles (155 nm) (153). Additionally, trafficking of liposome particles to lymph nodes has been shown to be size dependent, with small-sized vesicles (20–200 nm) freely draining to and specifically targeting lymph node-resident cells, while large-sized vesicles (500–2,000 nm) require dendritic cells for trafficking from the injection site to lymph nodes (154). More recently, immunization using a formulation containing large-sized cationic liposomes (~500 nm) resulted in enhanced splenocyte proliferative responses and reduced IL-10 production compared to small sized liposomes (~100 nm) (155). Interestingly, smaller unilamellar liposomes (70 nm) were reported to stimulate higher IgG titers than larger unilamellar (400 nm) but not large submicron size multilamellar liposomes in mice (156). The potency of multilamellar liposomes can be explained by more efficient antigen protection against degradation in their multiple lipid bilayers.

Liposomes can be modified to incorporate additional lipophilic immunomodulators within or attached to the lipid bilayer to enhance adjuvanticity. Such immunomodulators are crucial in the activation of the cells of the innate immune system via PRRs which recognize PAMPs on the surface of pathogens, subsequently activating the adaptive immune system. The activation of innate immune cells such as dendritic cells and macrophages requires the use of Toll-like receptor (TLR) and NOD-like receptor (NLR) type PRRs to direct a robust immune response (157). Therefore, the use of synthetic PRR agonists has been predicted to be critical in the formulation of liposome-based vaccine adjuvants (9, 158).

Liposome formulations can be customized by incorporating PRR agonists that mediate activation and maturation of APCs which in turn facilitates the uptake and processing of liposome-associated antigens resulting in potent cell-mediated immune responses (9). The most widely used PRR agonist monophosphoryl lipid A (MPLA), a TLR-4 agonist, has been used in licensed vaccine formulations Fendrix (hepatitis B) (159) and Cervarix (human papillomavirus) (160). A synthetic analog of MPLA, 3′-O-desacyl-4′-monophosphoryl lipid A formulated with *Quillaja saponaria* Molina, fraction 21 (QS-21) saponin is included in the liposome-based GSK Adjuvant System 01 (AS01) and has been tested in human studies for the malaria vaccine RTS, S (Mosquirix) (16), as well as a shingles sub-unit vaccine HZ/su which demonstrated over 90% efficacy amongst elderly persons (15). Similarly, liposomes can be tagged with sugars such as mannose to target them to lectin-like molecules on APCs to facilitate phagocytic uptake thereby promoting MHC class II involvement and, via cross presentation, MHC class I. This targeting of liposomes to different uptake pathways may aid in directing the resulting immune response toward a mixed Th1/Th2 response (9, 161).

The route of administration of particulate antigen delivery systems such as liposomes has been shown to affect the type and magnitude of immune response induced. Interestingly liposomes can be even administered orally; however, they need to be extensively modified to improve their stability in the gastrointestinal tract and their mucosa adhesive properties (162). In a cross-sectional study in mice, the intramuscular, intradermal and intralymphatic routes of administration were associated with intermediate to high induction of IgG2a and IFN-γ cytokine production while the subcutaneous route was associated with low elicitation of IgG2a and IFN-γ cytokine production (163). These data indicate that the route of administration is critical in the generation of the desired immune response and should be considered while interpreting immunological data following immunization with liposome-based vaccine formulations. Together, it is evident that the versatility and plasticity of liposomes facilitates the tailoring of the desired immune responses, as well as enhanced adjuvanticity and this can be exploited for the development of a malaria vaccine.

## Utility of Liposomes in Malaria Vaccine Development

Liposomes are increasingly becoming used in a number of malaria vaccine candidates targeting the different life-cycle stages. The use of liposomes in the development of sporozoite-stage malaria vaccines dates back to the mid-1980s where tetrapeptide antigens (asparagine-alanine-asparagine-proline) derived from the repetitive region of the circumsporozoite (CS) protein of *P. falciparum* sporozoites were conjugated to carrier proteins and were incorporated into liposomes. These studies demonstrated that liposomes containing carrier protein-conjugated peptide induced a potent humoral immune response which was further enhanced when lipid A was incorporated in the liposome formulation (164–169). The aforementioned studies laid the foundation for the development of RTS, S, the only vaccine against malaria that has received approval for use in pilot implementation trials in sub-Saharan Africa (7). The RTS, S vaccine construct is made of the central repeat region (amino acids 210-398) (R) and the C-terminal region containing the T-cell epitopes of CSP (T), fused to hepatitis B surface antigen (HBsAg) (S), co-expressed in *Saccharomyces cerevisiae* yeast and self-assembled with unfused HBsAg antigen (170, 171). These hybrid virus-like particles (VLPs) were co-formulated with GSK's proprietary liposome-based adjuvant system, AS01, which contains potent immunostimulants, MPLA and QS-21 that was selected over AS02, an oil-in-water emulsion adjuvant following evidence of enhanced antigen-specific antibody and CD4+ T-cell responses as well as improved efficacy in large-scale clinical studies (61, 172–175). The level of and mechanism of immunity induced by RTS, S in endemic settings are topics of much research. Over 4 years of follow up, the level of protection ranged from 18 to 36%, depending on the age of the child and whether the child received 3 or 4 doses of vaccine. Protection was clearly greater in the early months after vaccination, but waned rapidly after that and there was a ‘negative efficacy' during the 5th year in some children (176). This is a sub-optimal response and as such the mechanism of immunity came under great scrutiny. Evidence suggests that the level of antibody to the CS protein and serum levels of IFN-γ post vaccination both correlate with protection (177, 178). A major concern, has been the rapid diminution of antibody levels over time, in the face of parasite exposure. The problem is not that RTS,S is not immunogenic. The adjuvant system is one of the most potent there is for human use and is used elsewhere with great effect. It has made Shingrix a highly successful vaccine where even in the elderly there is 90% efficacy. Also, the problem is not with boosting *per se*, as each dose of RTS,S is accompanied by a rapid rise in antibody titer. In our view, the main problem is that even though RTS,S has a powerful adjuvant system, it is still not powerful enough. Titres wane after vaccination and natural infection will not boost. The main reasons for this appear to be: (i) antigenic polymorphism; and (ii) that the dose of sporozoites that individuals are exposed to (and which express the CS protein) are simply too low to boost or maintain antibody levels. T cell epitopes present on the CS protein which are incorporated into the vaccine are polymorphic (179), and this polymorphism does contribute to the low efficacy (180). The T-cell epitopes are known to be non-cross-reactive (181). An additional factor may relate to liposome design *per se*. Indeed, efforts are currently underway to remodel RTS, S such that each HBsAg particle expresses CSP thereby increasing the concentration of parasite antigens (182). Here, CSP-HBsAg fusion proteins were co-expressed in *Pichia pastoris* yeast which in the presence of a tightly regulated inducible alcohol oxidase (AOX1) promoter allows production of a higher density of hybrid VLPs (182, 183). This vaccine construct, co-formulated with Matrix-M ™, a saponin-based liposomal adjuvant is now undergoing clinical testing NCT02905019^1^.

In the development of transmission-blocking vaccines, gel core liposomes encapsulating Pfs25, an antigen expressed on zygotes and ookinetes of *P. falciparum* and a leading transmission-blocking vaccine (TBV) candidate, have been tested in mouse models (184). Gel core liposomes are a stabilized form of liposomes bearing a core of biocompatible polymer inside the lipid vesicle which serves to prevent the rapid release of antigen content from liposomes (184, 185). Following 2 intramuscular injections with gel core liposomes, Pfs25-specific antibody responses were observed in immunized mice, and these were maintained for up to 8 weeks. Additionally, strong Th1 cytokine (IL-2 and IFN-γ) responses were elicited and these responses were augmented when the gel core liposomes were formulated with CpG oligodeoxynucleotide (CpG-ODN) (184, 185).

A cationic adjuvant liposome formulation (CAF01) consisting of DDAB, synthetic mycobacterial cordfactor as an immunomodulator, and merozoite surface protein 1 (MSP1) antigen derived from *P. yoelii* genomic DNA, *Py*MSP1, has been tested in pre-clinical studies as a blood-stage malaria vaccine (186). Compared to the Alum adjuvanted vaccine formulation, immunization with CAF01- *Py*-MSP-1 resulted in significantly higher antibody and IFN-γ cytokine responses. Furthermore, following challenge, immunization with CAF01- *Py*MSP1 resulted in significant control of parasite growth (186).

Tyagi et al. (187), utilized a liposome-mediated transdermal immunization approach to deliver *P. falciparum* merozoite surface protein-1 (PfMSP-1) antigens through intact skin to antigen presenting cells in the skin (187). Similar to observations following immunization with CAF01- *Py*MSP1(186), durable and stronger parasite-specific humoral responses were observed up to 10 weeks post-immunization. Additionally, robust cell-mediated responses critical in immunity against blood-stage malaria parasites were induced following transdermal administration of elastic liposomes loaded with PfMSP-1 antigens when compared to Alum based vaccine formulations (187). Collectively, these data underscore the substantial superiority of liposome-based formulations over aluminum-based vaccine adjuvant formulations in the induction of parasite-specific immune responses to malaria.

More recently, our group formulated liposomes with mannosylated lipid core peptides (MLCPs) as targeting ligands for the delivery of whole blood-stage parasite antigens to professional antigen presenting cells (188). Immunization with these mannosylated liposome formulations resulted in the induction of significant CD8^+^ T cell responses; immunized mice demonstrated better control of parasitemia as well as extended survival following challenge, when compared with control mice, availing an alternative delivery system for inactivated whole parasite antigens (188). Together, the studies above indicate that liposomes are being considerably used in malaria vaccine development for targeting all of the different life-cycle stages and their pliability can be further explored to develop a multi-stage vaccine.

## Concluding Remarks

The quest for a vaccine against malaria continues despite the partial success with RTS, S/AS01, which showed modest efficacy in phase III clinical trials (16). Given the complex network of immune responses elicited following infection with *Plasmodium* parasites, an ideal vaccine should aim to induce the appropriate life-cycle stage-specific-antibody and cell-mediated responses capable of protecting against disease and immunopathology. The versatility of liposomes can be exploited to achieve an optimal formulation via the use of charged lipids to promote antigen retention at the injection site (depot-effect) (186), inclusion of targeting ligands to promote uptake by professional APCs (188), to control their stability, release of antigen, enhance antigen protection, and include immunomodulators (40, 43). Additionally, as the poor efficacy of most malaria vaccines evaluated thus far in field trials has been attributed to antigenic polymorphism, the use of whole parasite antigens (188) in liposome formulations needs to be explored further in malaria vaccine development. In summary, the modification of vesicle physicochemical properties may be further exploited to design an optimal liposome formulation with a high level of efficacy required for complete eradication of malaria by 2030 (8).

## Author Contributions

AS, DS, and MG drafted and reviewed original manuscript. AG, MZ, MS, and IT critically reviewed original manuscript and provided important intellectual content.

### Conflict of Interest Statement

The authors declare that the research was conducted in the absence of any commercial or financial relationships that could be construed as a potential conflict of interest.

## Footnotes

^1^A Safety and Efficacy Study of R21 +/- ChAd63/MVA ME-TRAP. https://ClinicalTrials.gov/show/NCT02905019.
